# Finding RNA structure in the unstructured RBPome

**DOI:** 10.1186/s12864-018-4540-1

**Published:** 2018-02-20

**Authors:** Yaron Orenstein, Uwe Ohler, Bonnie Berger

**Affiliations:** 1 Computer Science and Artificial Intelligence Laboratory, MIT, Cambridge, MA USA; 2 Max Delbruck Center for Molecular Medicine, -Buch, Berlin, Germany; 3 Mathematics Department, MIT, Cambridge, USA

**Keywords:** RNA-binding proteins, RBP, RBPome, RNA structure, eCLIP, RNAcompete

## Abstract

**Background:**

RNA-binding proteins (RBPs) play vital roles in many processes in the cell. Different RBPs bind RNA with different sequence and structure specificities. While sequence specificities for a large set of 205 RBPs have been reported through the RNAcompete compendium, structure specificities are known for only a small fraction. The main limitation lies in the design of the RNAcompete technology, which tests RBP binding against unstructured RNA probes, making it difficult to infer structural preferences from these data. We recently developed RCK, an algorithm to infer sequence and structural binding models from RNAcompete data. The set of binding models enables, for the first time, a large-scale assessment of RNA structure in the RBPome.

**Results:**

We re-validate and uncover the role of RNA structure in the RPBome through novel analysis of the largest-scale dataset to date. First, we show that RNA structure exists in presumably unstructured RNA probes and that its variability is correlated with RNA-binding. Second, we examine the structural binding preferences of RBPs and discover an overall preference to bind RNA loops. Third, we significantly improve protein-binding prediction using RNA structure, both in vitro and in vivo. Lastly, we demonstrate that RNA structural binding preferences can be inferred for new proteins from solely their amino acid content.

**Conclusions:**

By counter-intuitively demonstrating through our analysis that we can predict both the RNA structure of and RBP binding to these putatively unstructured RNAs, we transform a compendium of RNA-binding proteins into a valuable resource for structure-based binding models. We uncover the important role RNA structure plays in protein-RNA interaction for hundreds of RNA-binding proteins.

**Electronic supplementary material:**

The online version of this article (10.1186/s12864-018-4540-1) contains supplementary material, which is available to authorized users.

## Background

Protein–RNA interactions play vital roles in many processes in the living cell. These effect of a wide variety of cellular processes, including RNA replication, repair, recombination and post-transcriptional regulation [1]. More than 1500 genes in the human genome are thought to code for RNA-binding proteins (RBPs), making this set of human proteins one of the largest in the human proteome [2]. Most RBPs bind RNA through both sequence and structure. Thus, the ability to use RBP sequence- and structure-specific binding preferences would improve predictions of their role and function throughout the transcriptome.

Various high-throughput experimental techniques have been developed to measure protein-RNA binding. The CLIP-seq protocol and its variants measure protein-RNA binding in vivo, but they all suffer from noise and technological biases [3, 4]. In vitro methods, such as RNAcompete [5], provide a cleaner signal for the interactions without the confounding factors. Indeed, several methods have been developed to infer structural information from these data [6–8], but none were applied to the most comprehensive dataset of protein-RNA binding measurements, mainly due to the fact that the RNA probes were designed to be unstructured [9]. Just recently, we developed an algorithm, RCK, to infer sequence and structural binding preferences from this seemingly unstructured data [10]. While we generated binding models for the largest dataset to date, we did not analyze it for the role of RNA structure in the RBPome. It remained unclear how much structural information actually existed in the data, and how much downstream analysis of protein-RNA interactions would benefit from it. A previous study answered some of these questions, but was limited to a much smaller dataset of merely 30 proteins [11].

Here, we show, for the first time on a large scale, that RNA structural variability exists in unstructured RNA probes, and that it can be used to significantly improve the accuracy of binding predictions in various analyses. First, we show, via different approaches, that structure variability exists in the data, and that it correlates with protein binding. Then, we newly apply our recently-developed method RCK to catalog binding preferences of RNA-binding proteins on a large-scale. Third, we demonstrate how RNA-binding structural preferences learned in vitro improves binding prediction, both in vitro and in vivo. Lastly, we demonstrate that using RNA structure improves machine-learning methods for inferring RNA-binding preferences based solely on amino acid sequence. Taken together, these results further highlight the important role RNA structure plays in the RBPome.

## Results

### Structural variability in unstructured RNA probes

We first sought to test how much structure variability exists in unstructured RNA probes, which are the basis of the largest compendium of protein RNA-binding measurements [9]. Unstructured RNA probes were defined as those that are not likely to reside in low free energy conformations. But still, as expected, structural variability could exist. To gauge the putative structural variability, we calculated two RNA structure measures: (i) the likelihood of each probe to be ‘unstructured’; and (ii) the probability of each nucleotide to be base-paired. Since we are interested in RNA-binding proteins, which are assumed to bind contiguous k-mers [6], we measured the standard deviation of the average base-pairing probability of 5-mers. We applied these tests to the set of RNAcompete probes in comparison to a set of uniformly generated random probes of the same length.

**Fig. 1:**
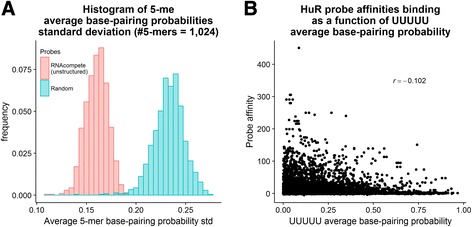
Structural variability exists in unstructured RNA probes and correlates with protein RNA-binding. **a**) Distribution of standard deviation of average 5-mer base-pairing probabilities. While in random probes there is more variability, unstructured probes are still quite variable. **b**) Correlation of probes containing UUUUU HuR-binding intensities and UUUUU’s average base-pairing probabilities. The negative correlation shows that HuR prefers to bind unpaired regions

Our results newly confirm on a large scale that unstructured probes are far from being unstructured. While the probes tend to have lower base-pairing probabilities than random probes, there exists significant structural variability in them. Indeed, more than 75% of unstructured probes have a non-zero probability of residing in a low-energy conformation, implying that some structure exists, albeit with smaller probability (Additional file 1: Fig. S1A). In terms of single-nucleotide base-pairing probabilities, unstructured probe probabilities are skewed towards zero more than random probes, but more than 90% of nucleotides have probability greater than 0 of base-pairing, spanning the range up to 1 (Additional file 1: Fig. S1B). This finding implies that unstructured probes likely contain many paired nucleotides. Moreover, when inspecting the average base-pairing probability of 5-mers, the standard deviation is centered at 0.16 (as compared to 0.23 for random probes), giving us a rough estimate for the expected base-pairing probability range of each k-mer (Fig. 1a).

To demonstrate that this variability is correlated with protein-binding affinity, we focused on the binding affinity of the well-studied HuR protein. The HuR protein is known to preferably bind uridine-stretches in unpaired RNA structural context [5]. Thus, we measured the average base-pairing probability of all UUUUU’s in RNAcompete probes, and compared their probability with the probes’ experimentally-measured binding affinity (Fig. 1b). The correlation is significantly negative, r = − 0.102 (*p*-value = 8.88·10^− 22^). Correlation of UUUUU base-pairing probabilities for random probes, using the same binding affinities, was r = 0.012 (*p*-value = 0.29). These results show that variability at RNA structure binding sites is correlated with experimental binding affinities, and can potentially aid in learning RNA sequence and structure binding preferences of RBPs. We note that the correlation might be so slight due to the low base-pairing probability and structural variability in the RNAcompete probes.

### RNA structural binding preferences for a compendia of RNA-binding proteins

We newly measured the distribution of structural preferences for a large compendium of RNA-binding proteins (RBPs) [9]. Thanks to the structural variability that exists in unstructured probes, we were recently able to develop software to infer RBP preferences from high-throughput data (i.e., RCK) [10]. It is commonly believed that almost all RBPs bind single-stranded RNA, but real evidence existed for only a few proteins [11]. Our structure-based binding models offer a novel way to analyze overall structural preferences in the RBP compendium.

**Fig. 2:**
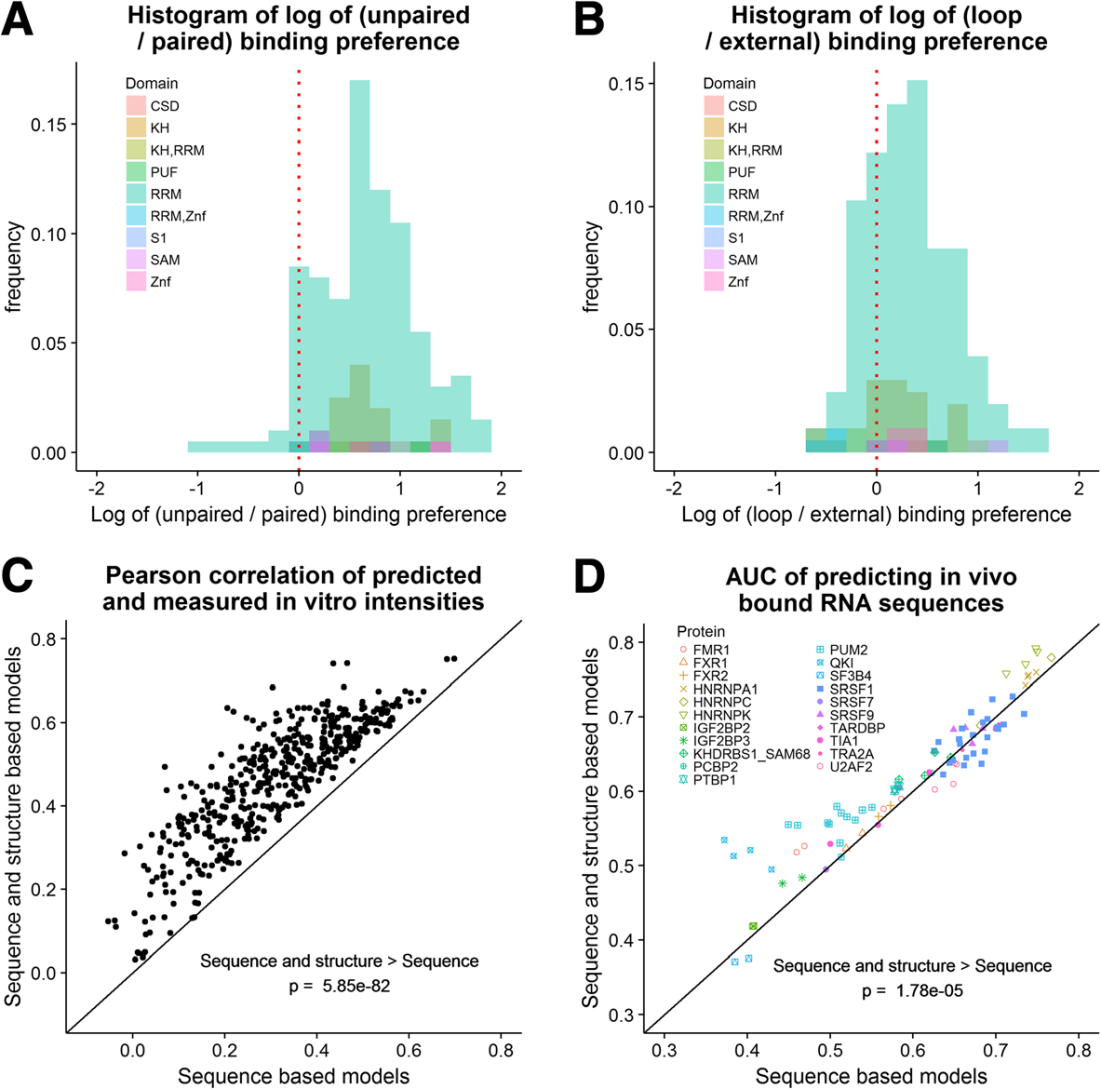
Analysis of RNA structural binding preferences for a large compendium of RBPs. **a**) Distribution of paired to unpaired binding preference log ratios for 205 RBPs shows almost all proteins in this dataset prefer to bind unpaired regions. **b**) Distribution of loop to external binding preference log ratios for 205 RBPs reveals more proteins in this dataset prefer to bind loop regions, and many may bind both. **c**) RNA structural binding preference improves in vitro binding prediction. Correlation results over 488 paired experiments uncovers that RNA structure plays a significant role in protein-RNA interactions. **d**) RNA structural binding preference improves in vivo binding prediction. AUC results of 96 paired eCLIP and RNAcompete experiments over 21 joint proteins demonstrate that RNA structural binding preferences learned from in vitro data correlate well with protein-RNA interactions measured in vivo

Here, we perform what we believe to be the first rigorous test of the assumption that many RBPs prefer to bind unpaired regions [11, 12]. For each protein, we calculated the log ratio of the binding score to the consensus sequence in an unpaired, relative to a paired, structural context. Our results indicate that almost all proteins in this dataset prefer to bind unpaired regions (Fig. 2a), a finding that supports the previous assumption that the majority of RBPs prefer to bind unpaired regions, as this dataset represents a wide variety of RBPs, and that RBPs which bind double-stranded RNA are an exception. The few proteins that prefer a paired context according to our binding models may either bind in a double-stranded context or result from errors in the model inference due to experimental noise.

While RBPs prefer to bind unpaired regions as a whole, some prefer to bind either loop or external regions; both regions are unpaired, yet loop regions are surrounded by a stem of paired nucleotides. To examine the preference of RBPs to bind loop or external regions, we measured the ratio of binding to loop regions as compared to external regions. Our results show that proteins vary in binding preference, and many do not demonstrate a strong preference (i.e., log ratio ranging from − 1 to 1); however, overall more proteins in this dataset prefer to bind loop regions than external regions (Fig. 2b). We observe no domain-specific preference for paired or unpaired contexts, nor for external or loop regions. This finding is likely due to the fact that the definition of these domains is broad: in the two largest domains, RNA recognition motif (RRM) and hnRNP K-homology (KH), the proteins within each domain share little sequence similarity [9].

### Improved predictive accuracy of in vitro and in vivo binding through RNA structure

We gauged the contribution of RNA structural preferences in binding prediction. We employed a comprehensive dataset of both in vitro and in vivo data. For in vitro prediction, we used 244 RNAcompete paired experiments, where we trained a model based on one half of a pair and tested on the other half of the same pair, resulting in 488 predictions [9]. Predictions were evaluated using Pearson correlation between predicted and measured probe intensities (as in [13]). For in vivo prediction, we used the available eCLIP data that covers 73 proteins, out of which 21 have a corresponding RNAcompete experiment [14]. We trained a model on a complete RNAcompete dataset and tested it on each eCLIP experiment on the same protein, resulting in 96 pairs of RNAcompete and eCLIP experiments. Predictions were evaluated using AUC (area under the ROC curve), where negative sequences were extracted from nearby regions. We repeated these tests with randomized structure probabilities to validate the significance of the addition of structure scores.

We found that adding structure preferences to the binding models significantly improves prediction performance, both in vitro and in vivo. In terms of in vitro binding, the improvement is across the board: for every single dataset, the performance improved by adding structure to the model (Fig. 2c). The improvement was striking, from an average Pearson correlation of 0.31 for sequence-only mode, to 0.46 when using sequence and structure (*p*-value = 5.85·10^− 82^, Wilcoxon rank-sum test). When we assigned random structure probabilities, this improvement disappeared (*p*-value = 1) (Additional file 2: Figure S2A). In vivo the improvement was more modest: from average AUC of 0.597 to 0.614 (*p*-value = 1.78·10^− 5^) (Fig. 2d), and similarly assigning random structure probabilities abolished this improvement (*p*-value = 0.829) (Additional file 2: Figure S2B). We can see several reasons for the dichotomy between in vitro and in vivo. The in vivo dataset is smaller— only 94 pairs compared to 488, covering only 21 proteins compared to 205. The in vivo experiments are known to be more noisy and prone to technological artifacts. The in vivo environment contains many confounding factors, such as competing and cooperative proteins and RNA degradation and expression. These are not part of the binding model, and thus may decrease prediction accuracy. Lastly, RNA secondary structure is less accurate for long sequences in vivo than for short sequences and in vitro [15]. Still, in some cases the addition of RNA structure increases AUC values from lower to higher than 0.5, showing that in extreme cases a protein may only bind in a specific structural context.

**Fig. 3:**
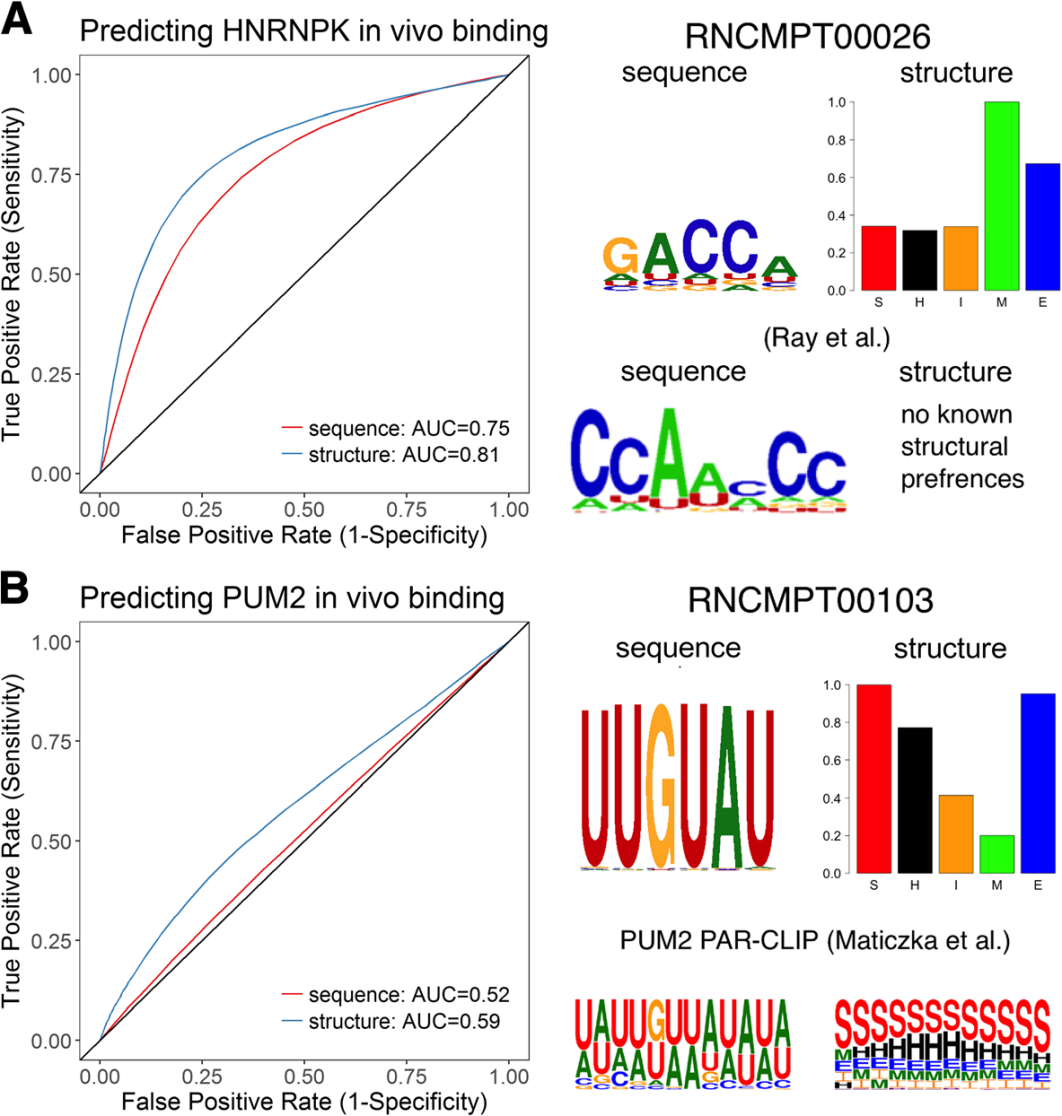
Structure-based models improve in vivo binding prediction. **a**) HRHNPK in vivo binding prediction improves AUC from 0.75 to 0.81. Sequence preferences agree with a previous study, but no structural preferences were previously known. **b**) PUM2 in vivo binding prediction improves AUC from 0.52 to 0.59. Sequence and structural preferences agree with previously-published preferences based on PAR-CLIP data inferred by GraphProt algorithm. Structural contexts letter: S = stem / paired, H = hairpin loop, I = inner loop, M = multi loop, E = external region

We specifically focus on two RBPs, HRNPK and PUM2, for which structural information contributed most to in vivo binding prediction accuracy. HNRNPK is an hnRNP family protein that plays diverse roles in multiple processes [16]. Ray et al. found that HNRNPK binds the sequence AGACCAA with highest affinity, but with no knowledge of its structural preferences [9]. RCK models inferred from the same RNAcompete data show that HNRNPK binds GACCA with the highest affinity, preferably in either loop or external unpaired regions (Fig. 3a). The increase in binding prediction accuracy in in vitro data was 0.196 in Pearson correlation, providing evidence for the importance of RNA structural preference in HNRNPK binding. The in vivo binding prediction based on the in vitro model improved the AUC from 0.75 to 0.81; that is, more real binding sites were predicted with fewer false positive sites with the addition of structural information (Fig. 3a), demonstrating concordance between in vitro (RNAcompete) and in vivo (eCLIP) experiments in terms of RNA-binding structural preferences.

The second protein assessed in the current study, PUM2, is a sequence-specific RNA-binding protein that acts as a post-transcriptional repressor [17]. According to our model, PUM2 prefers to bind UGUAA in a paired context; i.e., binds in a double-stranded context (Fig. 3b). Our finding was further confirmed by an independent method, GraphProt, on an independent dataset derived from a PAR-CLIP experiment [7]. The improvement in Pearson correlation on in vitro binding prediction is 0.14. The AUC for in vivo data improved from 0.52 to 0.57 (Fig. 3b), again demonstrating agreement between structural preferences measured in vitro and in vivo.

### Learning protein RNA-binding preferences from amino acid sequence

We explored the ability to predict RNA-binding preferences from amino acid sequence alone. This capability would enable binding prediction for mutated proteins (e.g. from genetic variation) as well as proteins with no experimental validation. In a recent study, Pelosoph et al. developed AffinityRegression, an algorithm for learning and predicting amino-acid k-mer associations with DNA and RNA k-mers [18]. These associations, which are family-specific, enable novel predictions of binding preferences for a specific protein from the amino acid content of its binding domain. The authors applied their method to the RNAcompete compendium and showed that they can predict sequence-binding preferences.

We sought to improve AffinityRegression’s performance by including RNA secondary structure in the model. We augmented the RNA k-mer space with structure-based probabilities based on five contexts: paired, hairpin loop, inner loop, multi loop and external. We applied the same tests as used by AffinityRegression to gauge performance: Pearson correlation between predicted probe intensities to the measured ones, and area under the precision-recall curve (AUPR) for ranking 1% of top probes compared to (i) 50% of bottom probes, and (ii) 99% of bottom probes.

**Fig. 4:**
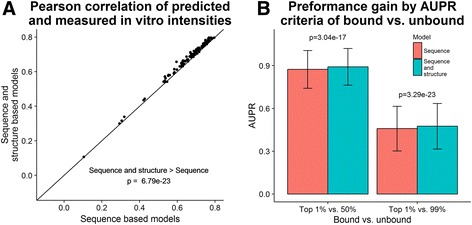
Improved binding prediction from amino acid sequence by utilizing RNA structure. **a**) When we add RNA structural features to the sequence k-mer space of AffinityRegression, we predict binding significantly better than using sequence features alone. **b**) When we add RNA structural features to the sequence k-mer space of AffinityRegression, we predict the top-bound probes as compared to unbound probes significantly better than using sequence features alone

We find that by augmenting the original models with RNA secondary structure, prediction accuracy improves significantly for the 130 proteins (Fig. 4a): the average correlation between predicted and real probe intensities increased from 0.688 to 0.705 by adding secondary structure (*p*-value = 6.79·10^− 23^ Wilcoxon signed-rank test). Performance also improved significantly when gauged by AUPR criteria (Fig. 4b): the average AUPR increased from 0.874 and 0.459 to 0.892 and 0.475 for 1% top probes vs. 50% and 99% bottom probes, respectively (*p*-values = 3.29·10^− 23^, 3.04·10^− 17^). When RNA structure probabilities are assigned randomly, we see no significant improvement when utilizing RNA structure (p-values = 0.985, 0.571, 0.915, referring to the correlation and AUPR criteria, respectively) (Additional file 3: Fig. S3).

There are a few reasons for the perhaps small but significant improvement. The sequence-only method is based on RNA 7-mer features. Since RNA structure is predicted from sequence, and most nucleotide base pairings are local, most of them are encoded by 7-mer features. Nevertheless, the improvement we demonstrate by adding secondary structure is significant and was observed for 126 out of 130 proteins tested. Moreover, the RNA probes were designed to be unstructured, and thus the information that can be extracted from the structural variability in them may be limited.

## Discussion

We have demonstrated the utility of RNA structure based protein binding models in various applications. We have shown that both sequence and structure binding models are accurately inferred from unstructured in vitro data. These models can further be used to enhance binding prediction, as we show both in vitro and in vivo. Moreover, they can improve prediction of binding preferences based on protein amino acid sequence.

In this study we analyzed the largest compendium of RNA-binding measurements to date, but it has a few limitations. Our analysis is restricted to binding models based on in vitro data and protein binding domains (as opposed to full-length proteins). While our models only address local RNA structure, and thus may miss more complex structures of long RNA molecules found in vivo, we were able to significantly improve in vivo binding prediction using structural information as opposed to using sequence scores alone. Detecting binding preferences to large RNA structures is still a major challenge as computational RNA structure prediction has been shown to be less accurate for long RNA molecules in vivo [15]. Still, the improvement in vivo demonstrates the biological relevance of our findings.

We wish to expand the RBPome prediction by augmenting it with new protein-RNA binding models and more accurate RNA structure predictions using experimental and computational advancements. We plan to expand our dataset of binding models by developing algorithms to learn RNA structure and sequence preferences from RNA bind-n-seq, a new technology to measure protein RNA-binding in vitro [19]. We also plan to take advantage of the recent machine learning breakthroughs of deep learning to improve prediction accuracy. We would like to incorporate in vivo data to better understand the structural binding preferences of long RNA molecules. Last, we will use RNA structure-probing data as a more reliable source of RNA structural information as compared to computational prediction.

## Conclusions

In this work we have taken the first steps in exploring the role of RNA structure in the RBPome on a large scale. We have demonstrated the importance of RNA structure in various applications of protein-RNA interactions. We expect the insights from this study and our resource to aid the research community in making significant advances in understanding the critical roles of protein-RNA interactions.

## Methods

### Structural analysis of RNA probes

We measured the structure of two RNA probe sets. One is the set of 241,357 RNAcompete probes, containing 219,990 unique sequences [9]. The second is a set of randomly generated 241,357 unique random sequences, all based on a position-independent uniform distribution of nucleotides. To measure the probability of a probe being unstructured, we followed the definition of Ray et al. [9]. We ran RNAshapes version 2.1.6 in the following way: “RNAshapes -s -c 70.0 -r -M 30 -t 1 -o 2 “[20]. The reported probability is one minus the sum of probabilities of structures below − 2.5 kcal/mol threshold. For base-pairing probabilities we ran RNAplfold: “RNAplfold -u 1 -W 80 -L 40 “[21]. Using these probabilities, we calculated average probabilities over all 1024 possible 5-mers, and the standard deviation of these averages. Finally, we plotted and calculated the correlation of 5-mer UUUUU average base-pairing probabilities and intensities of probes it appeared in according to the RNAcompete experiment RNCMPT00032 [9].

### Statistical analysis of protein RNA-binding structural preferences

We explored the overall statistic of structural binding preferences amongst the set of RNA-binding proteins in the RNAcompete dataset [9]. We used the set of learned models from the RCK website [10]. We identified for each protein the k-mer *w* and structural context *a* with the highest binding score. We calculated the binding scores for k-mer *w* in the other structural contexts. The relative weight of those scores are the structural preferences assigned to that protein. For Fig. 2a we calculated the log of the ratio of paired to unpaired scores. For Fig. 2b we calculated the log of the ratio of loop to external scores.

### In vitro and in vivo binding predictions

We tested the benefit of adding structure to the models for in vitro and in vivo binding prediction. We used the set of learned models from the RCK website [10]. For in vitro binding prediction we used the RNAcompete dataset, that included 244 RNAcompete experiments [9]. For each experiment we used the model trained on Set A and tested it on Set B. As in previous studies [10, 13], we performed clamping of outlier intensities. The performance was measured by Pearson correlation of predicted and measured intensities. For in vivo binding prediction we used eCLIP experiments [22]. 21 proteins overlapped between these two datasets and were covered by 36 RNAcompete experiments and 54 eCLIP experiments. For each eCLIP experiment, the bound peaks were used as positive sequences, and regions 300 nt downstream were used as controls. Structure prediction was performed using RNAplfold together with 150 nt flanking regions (as in previous studies [7, 10]), and only the middle 40 nt were used for prediction. Performance was gauged by area under the ROC curve. We repeated these tests with random assignment of structure probabilities to validate the significance of the improvement. For each position we generated five random integer numbers (using rand() of C programming language). We then normalized these numbers to a distribution by dividing each by their total sum.

### Regression of RNA-binding preferences from amino acid sequence

We used the original code of AffinityRegression with some modifications [18]. For the RNA feature matrix, we extended the 7-mer counts by the probability of each 7-mer being in each of the RNA structural contexts: paired, hairpin loop, multi loop, inner loop and external. We modified the training to be on one half of the set of RNA probes, while testing on the other half. We repeated this test using random structure probabilities, as described above.

## Additional files

Additional file 1: Figure S1 Structure in unstructured probes. **A**) Distribution of probe unstructured probabilities. Random probes are less likely to be unstructured, but more than 75% of unstructured probes have smaller-than-one probability of being unstructured. **B**) Distribution of nucleotide base-pairing probabilities. In random probes they are more skewed towards 1, but in unstructured probes they span the entire probability range of [0,1]. (PNG 52 kb)

Additional file 2: Figure S2 **A**) RNA structural binding preferences do not improve in vitro binding prediction when random structure probabilities are assigned. Correlation results over 488 paired experiments reveals that RNA structure does not improve binding prediction when structure probabilities are assigned randomly. **B**) RNA structural binding preferences do not improve in vivo binding prediction when random structure probabilities are assigned. AUC results of 96 paired eCLIP and RNAcompete experiments over 21 joint proteins demonstrate that RNA structural binding preferences learned from in vitro data do not correlate well with protein-RNA interactions measured in vivo when structure probabilities are assigned randomly. (PNG 85 kb)

Additional file 3: Figure S3 There is no improvement in binding prediction from amino acid sequence by utilizing RNA structure with random structure probabilities. **A**) When we add RNA structural features to the sequence k-mer space of AffinityRegression, but assign structure probabilities randomly, we do no predict binding any better than using sequence features alone. **B**) When we add RNA structural features to the sequence k-mer space of AffinityRegression, but assign structure probabilities randomly, we do not predict the top-bound probes as compared to unbound probes any better than using sequence features alone. (PNG 68 kb)

## Abbreviations

AUPR: Area under the precision-recall curve
KH: Hnrnp K-homology
RBP: RNA-binding protein
RRM: RNA recognition motif
