# Supplementary figures and images for: Finding RNA structure in the unstructured RBPome
Source: BMC Genomics. 2018 Feb 20;19:154. doi: 10.1186/s12864-018-4540-1 (PMC5819699; doi:10.1186/s12864-018-4540-1)

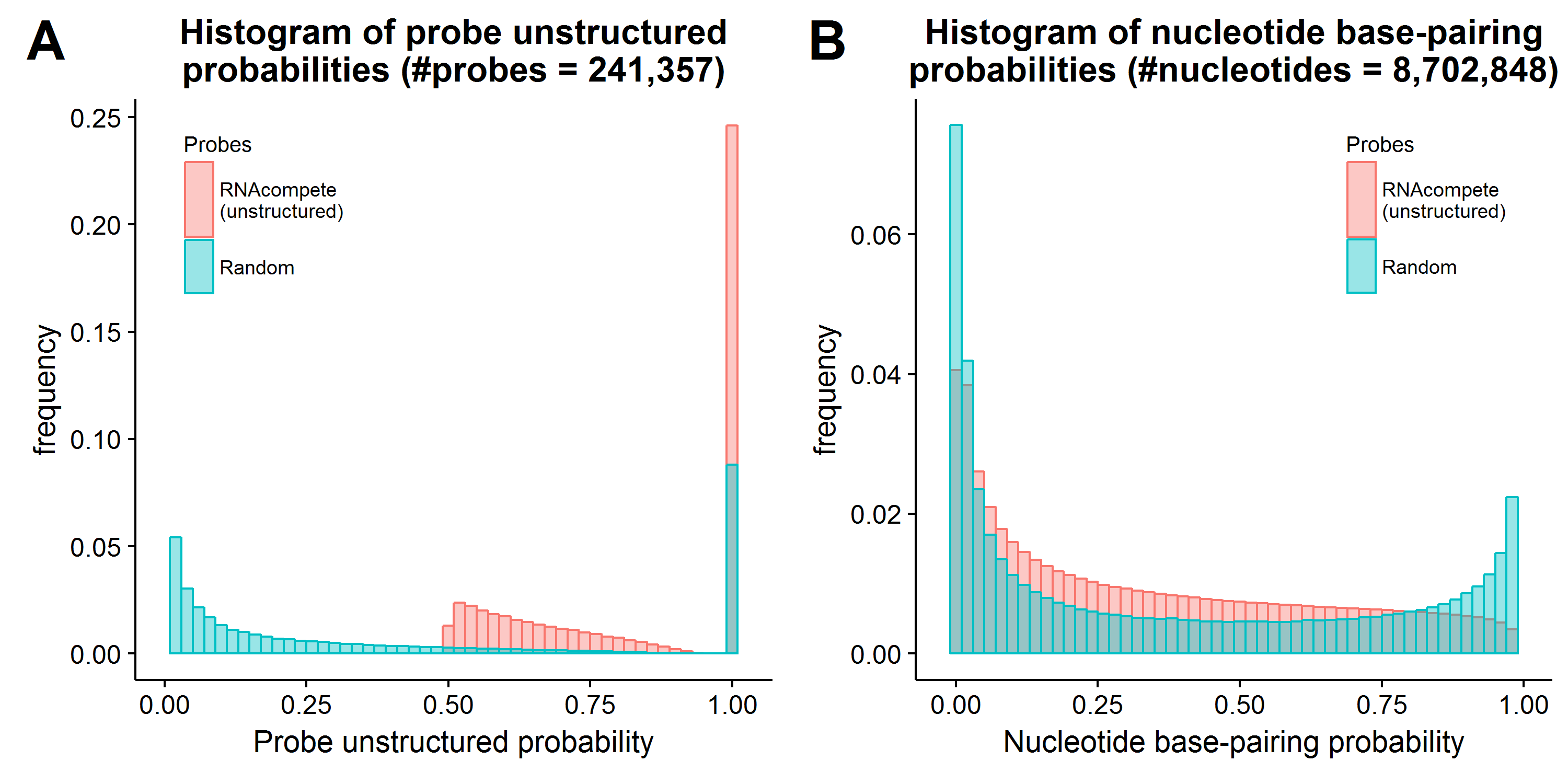

Supplement: Supplementary file 1 — Figure S1 Structure in unstructured probes. A) Distribution of probe unstructured probabilities. Random probes are less likely to be unstructured, but more than 75% of unstructured probes have smaller-than-one probability of being unstructured. B) Distribution of nucleotide base-pairing probabilities. In random probes they are more skewed towards 1, but in unstructured probes they span the entire probability range of [0,1]. (PNG 52 kb) [file 12864_2018_4540_MOESM1_ESM.png]

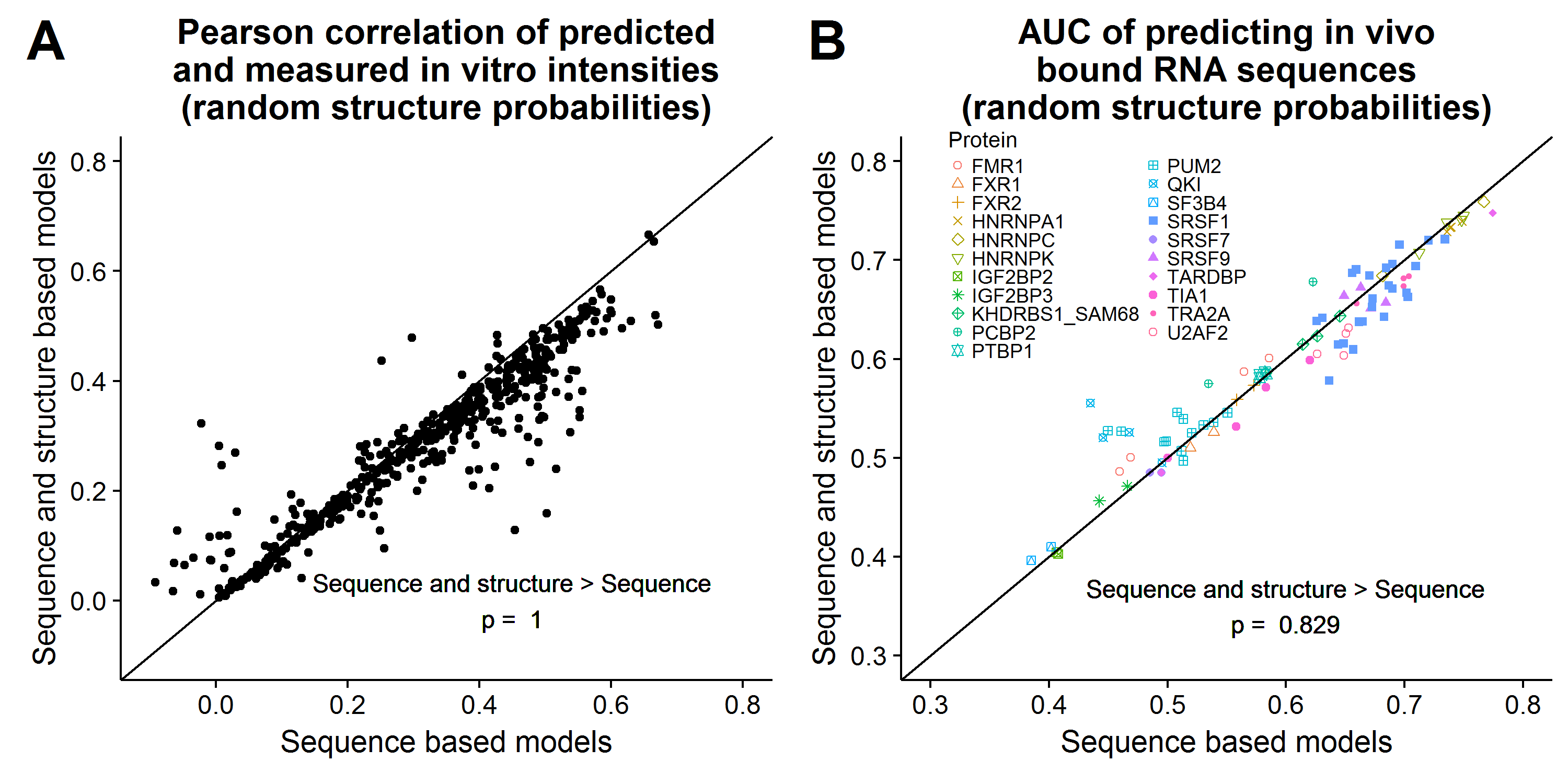

Supplement: Supplementary file 2 — Figure S2 A) RNA structural binding preferences do not improve in vitro binding prediction when random structure probabilities are assigned. Correlation results over 488 paired experiments reveals that RNA structure does not improve binding prediction when structure probabilities are assigned randomly. B) RNA structural binding preferences do not improve in vivo binding prediction when random structure probabilities are assigned. AUC results of 96 paired eCLIP and RNAcompete experiments over 21 joint proteins demonstrate that RNA structural binding preferences learned from in vitro data do not correlate well with protein-RNA interactions measured in vivo when structure probabilities are assigned randomly. (PNG 85 kb) [file 12864_2018_4540_MOESM2_ESM.png]

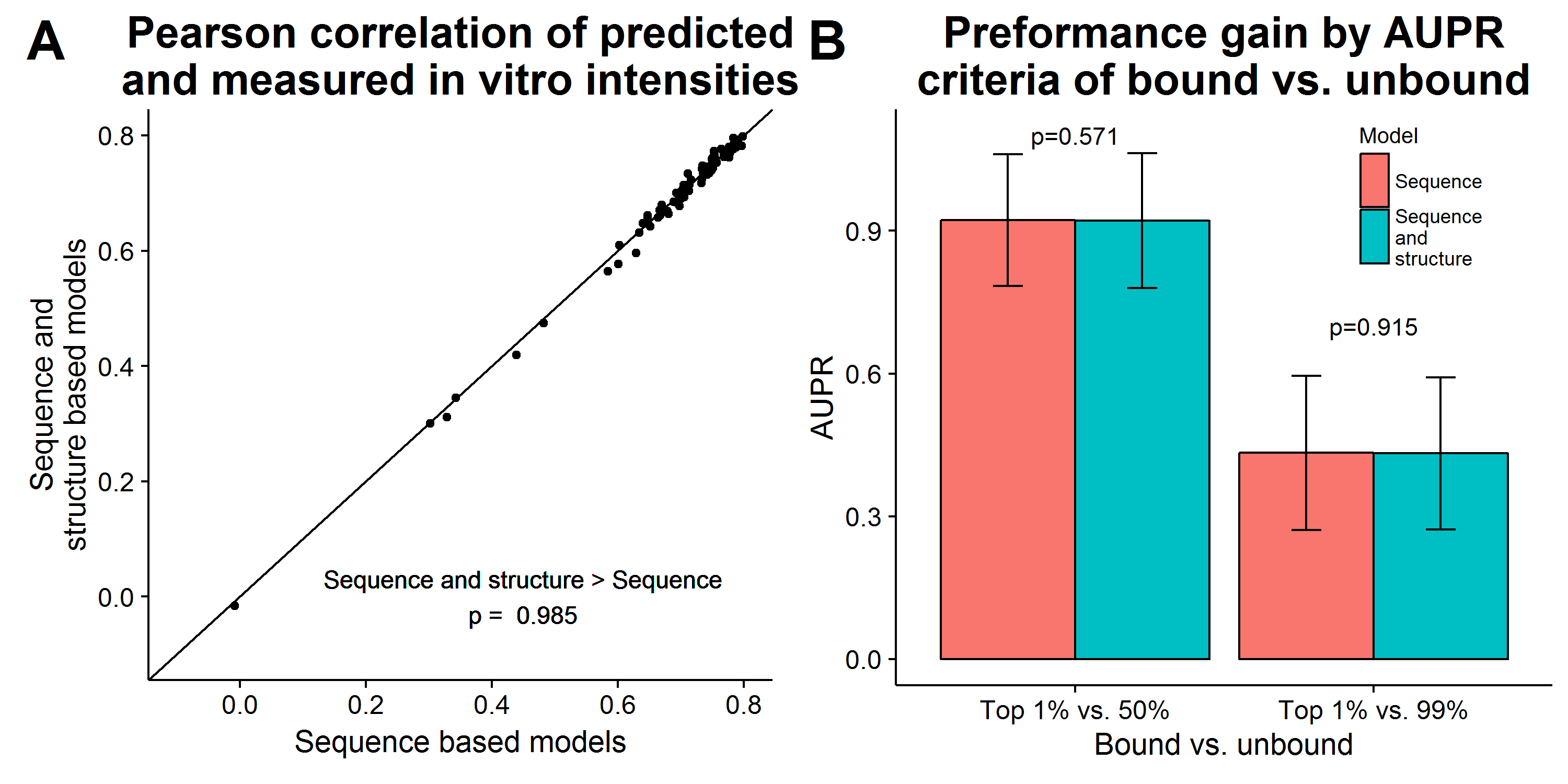

Supplement: Supplementary file 3 — Figure S3 There is no improvement in binding prediction from amino acid sequence by utilizing RNA structure with random structure probabilities. A) When we add RNA structural features to the sequence k-mer space of AffinityRegression, but assign structure probabilities randomly, we do no predict binding any better than using sequence features alone. B) When we add RNA structural features to the sequence k-mer space of AffinityRegression, but assign structure probabilities randomly, we do not predict the top-bound probes as compared to unbound probes any better than using sequence features alone. (PNG 68 kb) [file 12864_2018_4540_MOESM3_ESM.png]
